# Altering Intracellular Localization of the RNA Interference Factors by Influenza A Virus Non-structural Protein 1

**DOI:** 10.3389/fmicb.2020.590904

**Published:** 2020-11-12

**Authors:** Hua Wang, Zhonghui Tian, Yan Xu, Qi Wang, Shou-Wei Ding, Yang Li

**Affiliations:** ^1^State Key Laboratory of Genetic Engineering, School of Life Sciences, Fudan University, Shanghai, China; ^2^Department of Microbiology and Plant Pathology, University of California, Riverside, Riverside, CA, United States

**Keywords:** influenza A virus, non-structural protein 1 of influenza A virus, RNA-induced silencing complex, negative selection, pathogenicity, virulence of influenza A virus

## Abstract

Influenza A virus (IAV) causes seasonal infections and periodic pandemics in humans. The non-structural protein 1 (NS1) of IAV is the main viral antagonist of the innate immune responses that play a key role in influenza pathogenesis. However, the mechanism to disrupt the host cell homeostasis by IAV NS1 remains poorly understood. Here, we show that expression of NS1 from the WSN strain, but not PR8 strain, of IAV, markedly induced nuclear import of the host RNA interference (RNAi) factors such as Argonaute-2 and microRNA 16. We found that the single residue substitution of aspartic acid with histidine at position 101 (D101H) of IAV-PR8 NS1 was sufficient to induce the nuclear import process and to enhance the virulence of IAV-PR8 in mice. However, we observed no significant differences between the wild-type and mutant IAV-PR8 in virus titers or induction of the interferon response in lung tissues, indicating a novel role of NS1 in the virulence determination of IAV in a mammalian host. Moreover, our bioinformatic analysis of 69,057 NS1 sequences from all IAV subtypes deposited in the NCBI database revealed that the NS1-H101 gene of IAV-WSN was widespread among H1N1 viruses isolated in 1933 but disappeared completely after 1940. Thus, IAV NS1 (H101) is a mutation selected against during evolution of IAV, suggesting that mutation H101 confers an important biological phenotype.

## Introduction

Influenza A virus (IAV), an enveloped RNA virus belonging to the *Orthomyxoviridae* family, is one of the best-characterized viral pathogens (Krammer et al., 2018). It has worldwide prevalence and causes recurrent epidemics and occasional pandemics (Neumann et al., 2010; Ayllon and Garcia-Sastre, 2015b; Krug, 2015; Coughlan and Palese, 2018; Yamayoshi and Kawaoka, 2019). Unlike many other RNA viruses, IAV RNA replication and transcription occur in the nucleus of the infected cells (Hale et al., 2008; Rossman and Lamb, 2011; Tscherne and Garcia-Sastre, 2011). IAV contains an eight-segmented, negative-polarity RNA genome that encodes a maximal set of 14 proteins (Krammer et al., 2018). Among the viral proteins, non-structural protein 1 (NS1), which is expressed in the infected cells but not packaged in virions, has multiple well-characterized functions in viral infection, host innate/adaptive immune responses, and cellular signaling pathways, including blocking protein kinase R (PKR)-mediated inhibition of protein synthesis, interacting with the cellular protein phosphatidylinositol-3-kinase (PI3-kinase), blocking 2′-5′-oligoadenylate synthetase (2′-5′-OAS) activation of RNase L and limiting cytoplasmic helicase RIG-I (retinoic acid-inducible gene I) signaling (Hale et al., 2008; Ayllon and Garcia-Sastre, 2015a,b; Krug, 2015; Raman and Zhou, 2016).

MicroRNAs (miRNAs) are small, non-coding RNAs, typically 21 to 24 nucleotides (nt) in length (Carthew and Sontheimer, 2009; Kim et al., 2009). Primary miRNA transcripts (pri-miRNAs) are transcribed by RNA polymerase II and processed to precursor miRNAs (pre-miRNAs) in the nucleus by a microprocessor complex composed of DGCR8 and Drosha (Denli et al., 2004; Gregory et al., 2004; Gebert and MacRae, 2019). Pre-miRNAs are exported to the cytoplasm by exportin-5 (Yi et al., 2003; Bohnsack et al., 2004). In the cytoplasm, pre-miRNAs are processed by the RNase III enzyme Dicer into mature miRNAs (Bernstein et al., 2001; Hutvagner et al., 2001). Meanwhile, Dicer interacts with the transactivation response RNA-binding protein (TRBP) and the protein activator of PKR (PACT), which are critical for miRNA processing efficiency and specificity (Lee et al., 2006, 2013; Kok et al., 2007). Mature miRNAs are then loaded into an Argonaute (AGO) protein, an essential component of the RNA-induced silencing complex (RISC), which mediates the cleavage or translational suppression of the target mRNAs (Bernstein et al., 2001; Bartel, 2004; Carthew and Sontheimer, 2009). Mammals encode four AGO proteins, AGO1–4 (Dueck et al., 2012). AGO2 is the only AGO protein that retains the activity to cleave the target RNA (Liu et al., 2004; Meister et al., 2004).

Cellular miRNAs typically direct RISC-dependent gene silencing in the cytoplasm. A growing number of studies, however, suggest that components of the miRNA pathway, especially AGO2, are also found in the nucleus of mammalian cells (Robb et al., 2005; Gagnon et al., 2014; Catalanotto et al., 2016; Kalantari et al., 2016a,b; Liu et al., 2018; Gebert and MacRae, 2019). In addition, some mature miRNAs can reenter the nucleus following Dicer processing in the cytoplasm (Hwang et al., 2007; Foeldes-Papp et al., 2009). These findings suggest that there are some mechanisms for the nuclear transport of Argonaute proteins and miRNAs and that miRNAs may have biological functions after reentry into the nucleus (Kalantari et al., 2016a; Li et al., 2019; Shuaib et al., 2019). Meister et al. (2004) showed that nuclear miR-21 could modulate the cleavage of the target RNA, identifying a role of nuclear miRNA in RNA processing. In a subsequent study, Weinmann provided evidence that importin 8 (IPO8), which belongs to the karyopherin β family, can affect the nuclear localization of AGO proteins (Weinmann et al., 2009). Recently, a study provided additional confirmation that the IPO8-mediated cytoplasmic-nuclear shuttling of mature miRNAs is dependent on the combination of IPO8 and the AGO2 complex (Wei et al., 2014).

A significant amount of research has found substantial evidence for a role of the host RNAi pathway genes in mammalian viral infections (Guo and Steitz, 2014; Ojha et al., 2016; Trobaugh and Klimstra, 2017; Bernier and Sagan, 2018). For example, Dicer and Drosha are functionally important in inhibiting human immunodeficiency virus type 1 (HIV-1) replication in human cells and HIV-1 may suppress the expression of the polycistronic miRNA cluster miR-17/92 (Triboulet et al., 2007). The replication cycle of hepatitis C virus (HCV) utilizes the host miR-122, which binds to HCV RNA and enhances the stability and translation of the viral RNA and the accumulation of virus (Jopling et al., 2005). In addition, miR-21 is upregulated during HCV infection and contributes to host immune evasion by inhibiting the interferon-α signaling pathway (Chen et al., 2013). Dengue virus NS4B protein decreases the expression of a series of proteins involved in the host miRNA/RNAi pathway, including Dicer, Drosha, and AGO2 (Kakumani et al., 2013).

Available evidence also suggests IAV interactions with host RNA interference (RNAi) pathway genes in the process of coevolution between the virus and host (Ojha et al., 2016; Bernier and Sagan, 2018). Studies show that cellular miRNAs can affect influenza virus replication and pathogenesis through direct or indirect interactions (Makkoch et al., 2016; Gao et al., 2019). miRNA-binding sites have been identified in the genome of influenza A viruses, suggesting that some host miRNAs may inhibit the replication of influenza viruses (Song et al., 2010; Ma et al., 2012). Moreover, IAV infection can alter the expression of a series of host miRNAs, and this altered expression has been demonstrated to be closely related to innate immune responses (Buggele et al., 2012; Bakre et al., 2013). Recently, several studies have shown that the mechanism of antiviral RNAi is evolutionarily conserved in mammals (Li et al., 2013, 2017; Maillard et al., 2013; Qiu et al., 2017). We showed that antiviral RNAi is induced upon IAV infection and potently suppressed by IAV NS1 (Li et al., 2017).

MicroRNAs are small non-coding RNAs expressed by almost all metazoans and have key roles in gene expression, organism development, and cell differentiation. In this study, we show that the NS1 protein encoded by IAV strain A/WSN/1/33 (WSN) exhibited a unique activity to enhance the accumulation of several host RNAi factors in the nucleus of the infected human cells. Further analysis revealed a critical role for histidine at position 101 of the NS1 protein (NS1-H101) in increasing the nuclear accumulation of AGO2 and miRNAs and enhancing the *in vivo* virulence of IAV. Notably, we provide evidence for negative selection against NS1-H101 by a mechanism known as “purging by drift” during the evolution of IAV.

## Materials and Methods

### Cell Lines and Viruses

Human embryonic kidney cells (293T) and canine kidney cells (MDCK) were maintained in Dulbecco’s modified Eagle’s medium supplemented with 10% fetal bovine serum. Both cell lines were from ATCC. Influenza A/WSN/1/33 (H1N1) (abbreviated here as WSN), its NS1 deletion mutant, designated WSNΔNS1, A/Puerto Rico/8/34 (H1N1) (abbreviated PR8), Sendai virus, and pDZ-PR8 plasmids were kind gifts from Drs. Adolfo García-Sastre and Peter Palese.

### Generation of NS1 Mutant Viruses

Replacement of the NS segment of PR8 with the NS segment of WSN created the reassortant virus designated PR8 7 + 1. Individual point mutations were introduced into the NS segment of PR8 (D101H, A155T, and D189N of PR8 NS1 protein, see below) and the mutant viruses rescued by standard procedures using an eight-plasmid reverse genetics system (Hoffmann et al., 2000). WSN, PR8, PR8 7 + 1, and PR8 mutants containing point substitutions in NS1, and WSNΔNS1 were propagated in MDCK cells and an MDCK cell line stably expressing NS1 fused with green fluorescent protein (gift from Dr. Adolfo García-Sastre). As described (Li et al., 2010), virus titers were determined by plaque assays.

**Table:** 

**Definition of virus**	**Information**
PR8 7 + 1	NS segment of WSN and the other seven segments of PR8
PR8 D101H	NS fragment of PR8 with D101H mutation and the other seven segments of PR8
PR8 A155T	NS fragment of PR8 with A155T mutation and the other seven segments of PR8
PR8 D189N	NS fragment of PR8 with D189N mutation and the other seven segments of PR8

### Immunofluorescence Staining (IF)

Immunofluorescence staining assays were performed with 293T cells. The cells were transiently transfected with pIRESneo-FLAG/HA AGO2 expression plasmid (Addgene # 10822) using TransIT^®^-LT1 transfection reagent (Mirus, Madison, WI, United States) according to the supplier’s recommended protocol. After 8 h, cells were infected by WSN viruses. Twelve hours after infection, cells were washed with PBS and fixed in 4% formaldehyde for 15 min at room temperature (RT). Cells were washed again in PBS and permeabilized with 0.3% Triton X-100 for 10 min at RT. To reduce non-specific binding, cells were blocked in 10% normal goat serum for 30 min and washed in PBS. Cells were incubated overnight with primary antibodies specific for FLAG-tag, washed with PBS, and incubated for 2 h with appropriate secondary antibodies. Cells were washed three times in PBS at RT. Slides were mounted with coverslips. Confocal images of fluorescently labeled cells were acquired with an Olympus inverted confocal laser scanning microscope (Olympus FV1000). The microscope was equipped with Ar and HeNe laser lines at 488 nm to detect Alexa Fluor 488 and at 633 nm to detect Alexa Fluor 633, and a UV diode at 405 nm was used for DAPI detection.

### Co-immunoprecipitation Experiments

For co-immunoprecipitation experiments, 293T cells were washed twice with PBS and lysed in 1 ml lysis buffer (Cell Signaling Technology) per 1 × 10^6^ cells. Lysates were pre-cleared by centrifugation at 20,000 *g* for 10 min and supernatants were incubated with 40 μl anti-Flag Affinity Resin (GenScript, Nanjing, China) for 5 h at 4°C before washing three times with ice-cold wash buffer (IBA BioTAGnology). The precipitated complexes were used for the detection of specific proteins as described below.

### Western and Northern Blot Assays

Western blot analysis was performed as described previously with minor modifications (Li et al., 2008). After SDS PAGE, transfer proteins to nitrocellulose membranes (Bio-Rad, Richmond, CA, United States) and block with Tris-buffered saline containing 0.1% Tween-20 and 5% skim milk for 1 h at RT before the membranes were probed overnight at 4°C with primary antibodies. Antibodies to NS1 and NP were gifts of Dr. Yan Zhou, and antibodies to Dicer, GAPDH, SP1, and PACT (Santa Cruz Biotechnology, Santa Cruz, CA, United States), Ago2 (Active Motif LLC, Carlsbad, CA, United States), TRBP (Abnova, Taipei, Taiwan), and Ago1 and β-actin (Cell Signaling Technology, Beverly, MA, United States) were from commercial suppliers. NS1 was detected by alkaline phosphatase-conjugated anti-rabbit IgG (AnaSpec, San Jose, CA, United States) with BCIB/NBT premix solution (Sigma-Aldrich, St. Louis, MO, United States). For the detection of less abundant cellular proteins, HRP-conjugated anti-rabbit or anti-mouse IgG secondary antibodies (Thermo Fisher Scientific, Rockford, IL, United States) were used with an enhanced chemiluminescence reagent (Amersham Biosciences, Piscataway, NJ, United States). Northern blot analysis was performed as described previously (Li et al., 2017); RNA was extracted from cells 12 h after infection, and a chemical crosslinking protocol was used (Pall et al., 2007).

### Transfection, Infection, and Analysis of Nuclear and Cytoplasmic miRNA and Protein Abundance

To determine the effect of NS1 on the cytoplasmic and nuclear accumulation of RISC components, 293T cells were seeded in a 6-cm plate at a density of 2 × 10^6^ cells/plate one day before infection. Cells were inoculated with serum-free DMEM (mock), WSN, WSNΔNS1, Sendai, or PR8 as described (Li et al., 2008) and harvested 12 h after infection in 1 mL 1 × PBS by scraping and centrifuged at 300 × *g* for 3 min. Cytoplasmic and nuclear fractions were prepared essentially as described (Hwang et al., 2007) with minor modifications. Cell pellets were resuspended by gentle pipetting in 800 μL lysis buffer A [10 mM Tris (pH 8.0), 140 mM NaCl, 1.5 mM MgCl_2_, 0.5% Nonidet P-40] and incubated on ice for 5 min. After centrifugation at 1,000 × *g* for 3 min at 4°C, an equal volume of phenol (Ambion, Austin, TX, United States) was added to the supernatant for RNA and protein purification from the cytoplasmic fraction. Nuclei present in the pellets were washed twice with lysis buffer A and resuspended in 1 mL TRIzol for both protein and RNA extraction according to the supplier’s recommended protocol. The prepared RNA and protein isolates from each sample were used for Western and Northern blot analysis.

To determine the effect of NS1 on the cytoplasmic and nuclear accumulation of mature miRNAs, 293T cells were seeded in a 6-cm plate at a density of 2 × 10^6^ cells/plate and transfected with 4 μg of a pCMV-MIR expression plasmid using TransIT^®^-LT1 transfection reagent (Mirus, Madison, WI, United States) according to the supplier’s recommended protocol. Human miRNA expression plasmids MIR-16 and MIR-21 were purchased from OriGene (Rockville, MD, United States), and each contained the pre-miRNA with 200–300 nucleotides of up- and downstream flanking sequences amplified from human genomic DNA and cloned between the CMV promoter and a poly(A) tailing signal. Eight hours after transfection, cells were infected by WSN or WSNΔNS1 (MOI = 3) as described and harvested 12 h later in 1 mL 1 × PBS to prepare total, cytoplasmic, and nuclear RNA fractions as described above. Half of the total yield of RNA from total, nuclear, and cytoplasmic fractions of each cell sample was used for Northern blot detection of mature miRNAs as described. To ensure successful subcellular fractionation, the same fractions were probed for a mitochondrion (Mito) tRNA-Val and U6 RNA, which are localized in the cytoplasm and nucleus, respectively.

### Plaque Assay and Virus Growth Curves

To determine IAV titers in cell supernatants, plaque assays were performed on canine kidney (MDCK) cells, and plaques were counted 2–3 days post infection (d.p.i.). Multiple-cycle growth curves of the WT and recombinant mutant viruses were analyzed in MDCK cells. Cells were infected by WT and recombinant mutant viruses of infection (MOI) of 0.001, the supernatant was harvested at the indicated time points, and virus titers were determined by plaque assays.

### Animals

Animals were obtained from Charles River Laboratory (Shanghai, China). All animals were maintained on the genetic background of BALB/c, and all experiments were performed with age (6–8 weeks old)- and sex (female)-matched mice. All the animal experiments in China were carried out under the guidelines of the Institutional Animal Care and Use Committee, Fudan University of China. Mice were reproduced and maintained in the specific pathogen-free animal facility at the College of Life Sciences, Fudan University.

### *In vivo* Influenza A Virus Infection

Animals were anesthetized and challenged by the intranasal administration of 2 × 10^4^ PFU of infectious virus in PBS or an equal volume of PBS. Mice were observed and monitored daily for weight loss and mortality for 8 days. Normally, we assumed that mice die by default when their weight dropped by 25%.

### Histopathology and Immunohistochemistry (IHC)

The lungs of BALB/c mice inoculated intranasally with PR8 WT, PR8 D101H, or PBS were harvested 3 d.p.i., fixed in 10% neutral-buffered formalin, transferred to 70% alcohol, and embedded in paraffin. For histological assessment, we subjected lung slices to hematoxylin and eosin staining and used light microscopy (NIKON ECLIPSE CI). For IHC, the slides were incubated overnight at 4°C with IAV NS1 mouse monoclonal antibody (Santa Cruz Biotechnology) at a dilution of 1:100 or IAV M1 rabbit polyclonal antibody at a dilution of 1:10,000 after antigen retrieval and blocking steps. After rinsing with PBS three times, the slides were incubated with HRP-conjugated secondary antibodies (Service bio) for 50 min at room temperature. After further washing, the sections were stained with 3,3′-diaminobenzidine (DAB) (DAKO). Finally, slides were counterstained with hematoxylin and observed by light microscopy (NIKON ECLIPSE CI).

### RNA Isolation, Reverse Transcription, and qPCR

Lungs were harvested from infected BALB/c mice 3 d.p.i. (*n* = 5 mice per group). Total RNA was isolated from homogenized lung tissue utilizing TRIzol reagent (Invitrogen) according to the manufacturer’s protocol. RNA was reverse transcribed using Reverse Transcription Supermix (Bio-Rad). The qPCR was performed using SYBR Green (Bio-Rad). All experimental samples were analyzed in triplicate. The relative gene expression was normalized to the housekeeping gene β-actin. Data were analyzed by the comparative CT method (ΔΔCT).

Primer sequences are as follows:

β-actin forward ATTGGCAACGAGCGGTTCC,

reverse AGCACTGTGTTGGCATAGAGG;

HA forward AGCAAAAGCAGGGGAAAAT,

reverse TGTTCGCATGGTAGCCTATAC;

IFN-β forward CACAGCCCTCTCCATCAACTA,

reverse CATTTCCGAATGTTCGTCCT;

RIG-I forward GAGAGTCACGGGACCCACT,

reverse CGGTCTTAGCATCTCCAACG;

Mavs forward CTGCCTCACAGCTAGTGACC,

reverse CCGGCGCTGGAGATTATTG.

### Sequence Retrieval and Bioinformatics Analysis

The influenza data were downloaded from Influenza Virus Resource^1^.

The homology search tool BLAST was downloaded from the NCBI. Multiple-sequence alignment was performed with Clustal Omega using the standard parameters. A neighbor-joining (NJ) tree was constructed using the Mega X program. Other analysis was performed by in-house scripts.

### Statistical Analysis

All statistical analyses were performed using Prism 8.0 (GraphPad Software, La Jolla, CA, United States). Survival curves were prepared via the product-limit method of Kaplan and Meier, and comparisons were made using the log-rank test. The qPCR assay data were analyzed by one-way analysis of variance (ANOVA) or by an unpaired *t*-test of biological replicates. In all cases, the differences were considered to be statistically significant at *p* value less than 0.05.

## Results

### The Activity of Influenza Viral NS1 to Induce Subcellular Redistribution of AGO2 and Mature miRNAs

**FIGURE 1:**
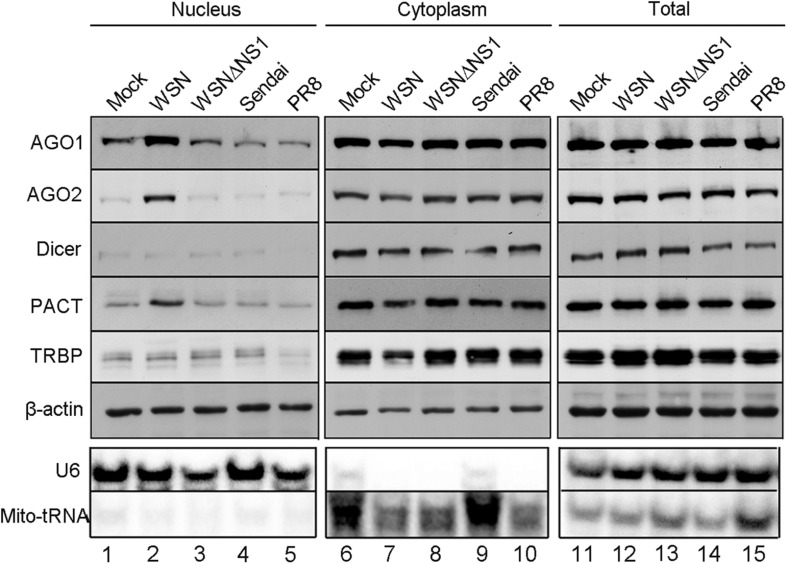
NS1 protein of WSN induces subcellular redistribution of RNAi factors in infected cells. Western and Northern blotting detection of RNA interference protein components and control RNAs in nuclear, cytoplasmic, and total extracts isolated from 293T cells 12 h after inoculation with buffer (mock), WSN, WSNΔNS1, Sendai, and PR8 viruses. Fractionation controls include β-actin, Mito-tRNA (cytoplasmic RNA), and U6 snRNA (nuclear RNA). Each experiment was repeated at least three times independently.

In an effort to understand RNAi suppression by the influenza A viral NS1 protein, we analyzed the subcellular accumulation of five RNAi components in human 293T cells after infection with either PR8 or WSN isolate of IAV. Cytoplasmic and nuclear fractions were isolated for Western blot analysis from 293T cells 12 h post infection with IAV-PR8, IAV-WSN, or Sendai virus as a control, which also contains a negative-strand RNA genome. We found no obvious differences in the total accumulation levels of Dicer, AGO1, AGO2, PACT, or TRBP in 293T cells after infection with IAV-PR8, IAV-WSN, or Sendai virus (Figure 1, right panel). However, we detected a markedly enhanced accumulation of AGO1, AGO2, and PACT, but not Dicer or TRBP in the nucleus of 293T cells after infection with IAV-WSN compared to mock infection or infection with IAV-PR8 or Sendai virus (Figure 1, lane 2). Notably, no obvious changes in the subcellular accumulation of AGO1, AGO2, and PACT were observed in 293T cells after infection with the mutant of IAV-WSN deficient in the expression of NS1 (WSNΔNS1, Figure 1, lane 3). As expected, abundant accumulation of Mito-tRNA and U6 snRNA was detected in the cytoplasmic and nuclear fractions of 293T cells, respectively (Figure 1). These findings reveal an activity of IAV-WSN NS1 to induce subcellular redistribution of AGO1 and AGO2 as well as PACT in the infected cells.

**FIGURE 2:**
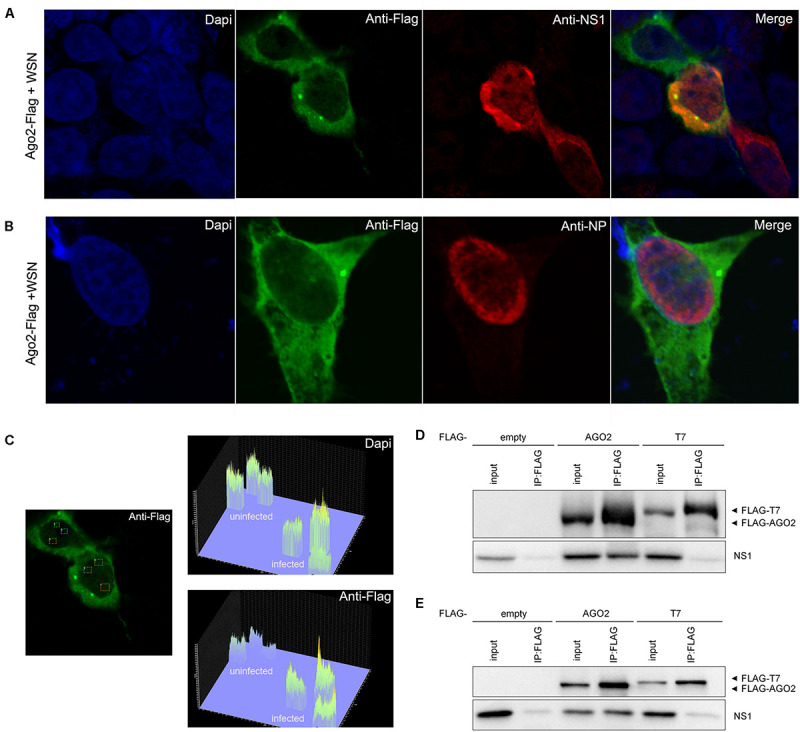
WSN NS1 protein presents in a complex with AGO2 in the 293T cells. Immunofluorescent analysis of 293T cells ectopically expressing Flag-AGO2 with WSN infection. **(A)** Flag-AGO2 and antibody-stained viral NS1 are co-localized in the infected cells. **(B)** Flag-AGO2 and antibody-stained viral NP are not co-localized in the infected cells. **(C)** Fluorescence intensity of nuclear DAPI and AGO2 in virus-infected or uninfected cells in **(A)**. FLAG-tagged epitope representing AGO2 labeling with AlexaFluor488 (Green). Viral NS1 **(A)** and NP **(B)** labeling with AlexaFluor633 (Red). Nuclei counterstaining with DAPI (Blue). Immunoprecipitation of WSN NS1 with AGO2 protein. **(D)** 293T cells were co-transfected with a plasmid encoding WSN NS1 plus an empty plasmid or a plasmid encoding Flag-tagged AGO2 or Flag-tagged T7. **(E)** 293T cells were transfected with a plasmid encoding Flag-tagged AGO2, Flag-tagged T7, or empty vector before infection by IAV-WSN. Immunoprecipitations were performed with anti-FLAG antibody. Flag-tagged proteins and NS1 were detected with specific antibodies.

We next performed an immunofluorescence staining assay to investigate the subcellular localization of AGO2 in 293T cells after IAV-WSN infection. Following IAV-WSN infection, we detected a strong fluorescence signal of AGO2 in the nucleus of infected 293T cells ectopically expressing Flag-AGO2 (Figures 2A,B). Interestingly, we found obvious co-localization of NS1 and AGO2 in the cytoplasm and nucleus of 293T cells after IAV-WSN infection (Figure 2A and Supplementary Figure S1). In contrast, co-localization was not detectable between AGO2 and the viral nucleocapsid protein (NP), which largely accumulates in the nucleus (Figure 2B). Quantitative comparison of fluorescence from random nuclear regions in the infected and uninfected cells in Figure 2A revealed that Flag-AGO2 fluorescence intensity in the nuclei of infected cells was significantly higher than that in the nuclei of uninfected cells (Figure 2C, top left, uninfected cell; middle, infected cell). Furthermore, we performed co-immunoprecipitation (co-IP) analysis in 293T cells expressing Flag-AGO2 protein. The results showed that NS1 was detected in AGO2 immunoprecipitants from cells ectopically expressing WSN NS1 and cells infected with IAV-WSN (Figures 2D,E). These results suggested that the NS1 protein may be present in a complex with AGO2 in the 293T cells infected with IAV-WSN.

**FIGURE 3:**
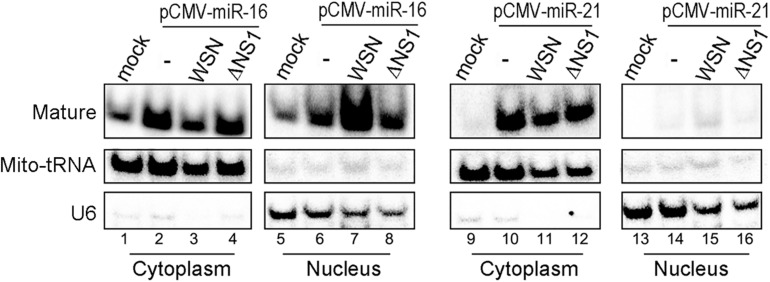
WSN NS1 induces nuclear importing of miRNAs in infected cells. Northern blotting detection of miR-16 or miR-21 in cytoplasmic and nuclear fractions from 293T cells transfected with a miRNA expression plasmid and infected by buffer (−), WSN or WSNΔNS1 viruses. Fractionation controls include Mito-tRNA (cytoplasmic RNA) and U6 snRNA (nuclear RNA). Each experiment was repeated at least three times independently.

We further compared the effect of infection with IAV-WSN or WSNΔNS1 on the cytoplasmic and nuclear accumulation of exogenous miR-16 and miR-21 in 293T cells from a transfected plasmid. The plasmids directed the overexpression of pre-miR-16 or pre-miR-21 plus 200–300 nucleotides up- and downstream flanking sequences amplified from human genomic DNA and cloned between the CMV promoter and a poly(A) tailing signal. Similar to some miRNAs (Gebert and MacRae, 2019), miR-16 was detectable in both the cytosol and nucleus of 293T cells without transfection with the overexpressing plasmid (Figure 3, lanes 1 and 5). However, we observed a dramatically increased accumulation of miR-16 in the nucleus of 293T cells infected with IAV-WSN compared to mock infection or WSNΔNS1 infection (Figure 3, compare lane 7 with lanes 6 and 8). In contrast, miR-21 remained predominantly in the cytosol after infection with either IAV-WSN or WSNΔNS1 (Figure 3, compare lanes 10–12 with lanes 14–16). Taken together, these results indicate that NS1 protein from IAV-WSN has the ability to alter the intracellular localization of several RNAi factors.

### Induced Subcellular Redistribution of AGO2 Requires Histidine at Position 101 (H101) of NS1

**FIGURE 4:**
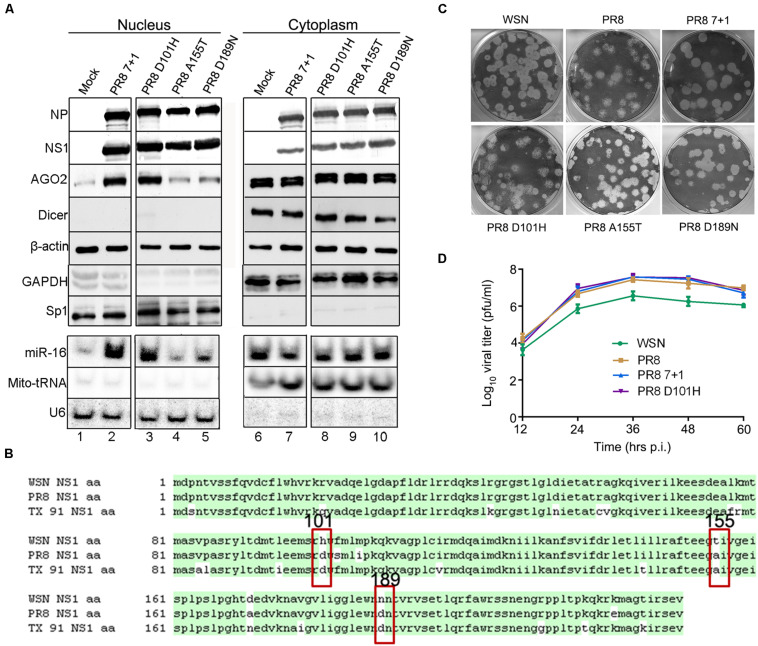
Induction of AGO2 subcellular redistribution requires histidine at position 101 (H101) of NS1. **(A)** Western and northern blotting detection of RNAi protein components and miR-16 in nuclear and cytoplasmic extracts isolated from 293T cells 12 h after inoculation with buffer (Mock), PR8 7 + 1, PR8 D101H, PR8 A155T, or PR8 D189N viruses. Fractionation controls include β-actin, GAPDH (cytoplasmic protein), Sp1 (nuclear protein), Mito-tRNA (cytoplasmic RNA), and U6 snRNA (nuclear RNA). **(B)** Amino acid sequence alignment of the NS1 proteins of influenza A virus WSN, PR8, and TX 91; three non-conservative amino acids are labeled at positions 101, 155, and 189. **(C)** Representative plaque assay images of the WT and recombinant mutant viruses on MDCK cells. **(D)** Multiple-cycle growth curves of the WT and recombinant mutant viruses on MDCK cells. Cells were infected by WT and recombinant mutant viruses of the same titer. Every 12 h post infection (h.p.i.), the virus titers were determined by plaque assay and quantified as plaque-forming units (p.f.u.) per ml. Mean titer values at each time point were plotted. Each experiment was repeated at least three times independently.

To further investigate the role of IAV-WSN NS1 in the subcellular redistribution of AGO2, we first rescued a reassortant virus, designated PR8 7 + 1. PR8 7 + 1 contained 7 of the 8 genome segments from IAV-PR8 with the smallest segment from IAV-WSN, which encodes NS1 and NS2 (also known as nuclear export protein) (Hale et al., 2008). Cell fractionation analysis showed that PR8 7 + 1 infection induced a markedly enhanced nuclear accumulation of AGO2, but not Dicer, in the infected 293T cells (Figure 4A lane 2 and Supplementary Figure S2). Thus, PR8 7 + 1 was similar to IAV-WSN and differed from IAV-PR8, indicating that the enhancement of AGO2 nuclear accumulation is determined by the NS segment of IAV-WSN.

**FIGURE 5:**
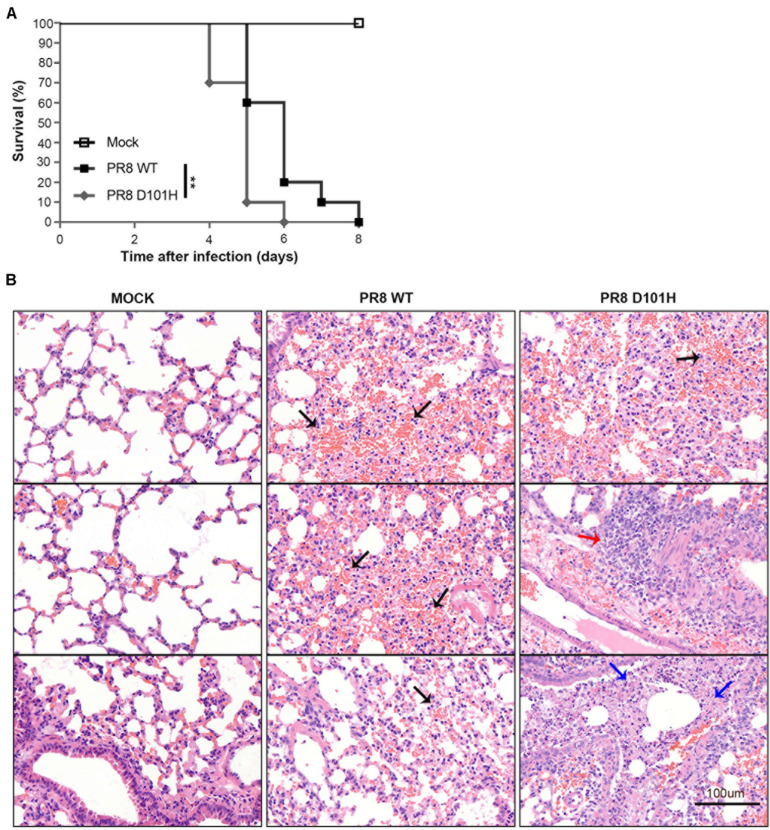
H101 mutation of NS1 contributes to viral pathogenesis in mice. **(A)** The survival of BALB/c mice was monitored. Weight loss of BALB/c mice was measured daily after intranasal inoculation with Mock, PR8 WT, or PR8 D101H virus (**p < 0.01; log rank). Mock infected (*n* = 5); PR8 WT infected (*n* = 10); PR8 D101H infected (*n* = 10). **(B)** Representative lung H&E slice images of BALB/c mice after intranasal inoculation with Mock (left panel), PR8 WT (middle panel), or PR8 D101H virus (right panel). Lungs were harvested 3 days after infection. Sections through the main bronchiole of the left lobe were stained with hematoxylin and eosin. Black arrows indicate hemorrhage, red arrow indicates inflammation, and blue arrows indicate necrosis. Scale bar: 100 μm.

IAV-WSN and IAV-PR8 are both H1N1 subtype strains originally isolated in the United Kingdom in 1933 and Puerto Rico in 1934, respectively, and their NS1 proteins differ by only 7 amino acids (Figure 4B). We found that infection with H1N1 A/Texas/36/91 (TX 91) did not alter AGO2 subcellular redistribution (data not shown), which further narrowed down to the three non-conserved amino acids that might be responsible for the phenotype of IAV-WSN NS1 (Figure 4B). Thus, we performed site-directed mutagenesis and rescued three mutants of IAV-PR8, PR8-NS1 (D101H), PR8-NS1 (A155T), and PR8-NS1 (D189N), each of which encoded a single amino acid substituted with that found in IAV-WSN NS1 at positions 101 (Asp/D to His/H), 155 (Ala/A to Thr/T), and 189 (D to Asn/N), respectively. Cell fractionation analysis showed that similar to IAV-PR8, infection with neither PR8-NS1 (A155T) nor PR8-NS1 (D189N) enhanced nuclear accumulation of AGO2 (Figure 4A lane 4,5). In contrast, PR8-NS1 (D101H) infection enhanced nuclear accumulation of AGO2 (Figure 4A lane 3), which was thus similar to the infection with IAV-WSN or PR8 7 + 1. However, we detected no obvious changes in the accumulation of the control nuclear and cytoplasmic proteins or RNAs.

Furthermore, we found that endogenous miR-16 accumulated to much higher levels in the nucleus of 293T cells following infection with PR8 7 + 1 or PR8-NS1 (D101H), but not PR8-NS1 (A155T) or PR8-NS1 (D189N) (Figure 4A lanes 2, 3). The plaque morphology of PR8-NS1 (D101H), PR8-NS1 (A155T), and PR8-NS1 (D189N) was indistinguishable from each other or from IAV-PR8 and IAV-WSN (Figure 4C). We also found no obvious differences in the growth curves of PR8 7 + 1, PR8 D101H, and wild-type IAV-PR8 (Figure 4D). These results indicate that the single amino acid substitution introduced into NS1 of IAV-PR8 is sufficient to induce the subcellular redistribution of AGO2 and miR-16 in the infected cells.

### H101 Mutation of NS1 Contributes to Viral Pathogenesis in Mice

We next compared the *in vivo* infection of mice with IAV-PR8 and PR8-NS1 (D101H), which differed by the single amino acid at position 101 of NS1. As expected, intranasal inoculation of mice with 2 × 10^4^ PFU mouse-adapted IAV-PR8 induced prominent signs of infection, such as weight loss, ruffled hair, hunched back, heavy breathing, and mortality (Figure 5A). We found that infection with the same dose of PR8-NS1 (D101H) caused significantly earlier mortality compared with IAV-PR8 infection (Figure 5A). Sequence analysis of PR8-NS1 (D101H) recovered from the lung tissues of infected mice found that the introduced mutation was stably maintained and no reversion to the wild-type sequence was detected.

To compare the progression of lung damage induced by the two viruses, we euthanized mice at 3 dpi and harvested the lung tissues to assess histological changes by hematoxylin–eosin staining of tissue sections from the left lobe (Figure 5B). Visualization of whole lung sections showed evidence of hemorrhaging in multiple regions of the lobe from mice infected with IAV-PR8. By comparison, infection with PR8-NS1 (D101H) induced enhanced inflammatory cell infiltration in the areas around the lung vasculature. We also observed multiple necrotic lesions in the lungs of the mice infected with PR8-NS1 (D101H), and most of the necrosis was adjacent to bronchioles or small bronchioles (Figure 5B).

**FIGURE 6:**
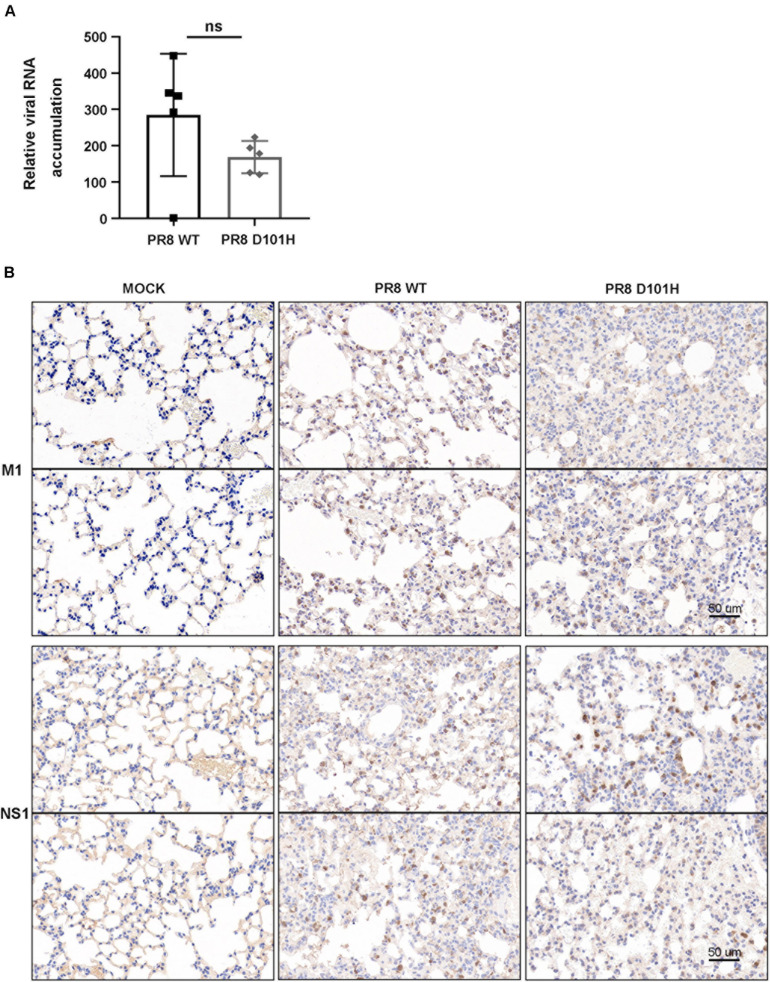
No significant difference in the accumulation of the two viruses in lung tissue. **(A)** Influenza viral titers were determined by qPCR in the lungs of BALB/c mice infected with PR8 WT and PR8 D101H viruses. Lungs were harvested 3 days after infection. Virus titers were quantified by relative HA expression. Differences between groups were analyzed by an unpaired *t*-test (ns, not significant). **(B)** Representative lung IHC slice images of BALB/c mice intranasally inoculated with Mock (left panel), PR8 WT virus (middle panel), or PR8 D101H virus (right panel). Lungs were harvested 3 days after infection. Viral infection was confirmed by immunohistochemistry with influenza A virus M1 polyclonal antibody (upper panel) and NS1 monoclonal antibody (lower panel). Sections through the main bronchiole of the left lobe were stained with antibody. Scale bar: 50 μm.

**FIGURE 7:**
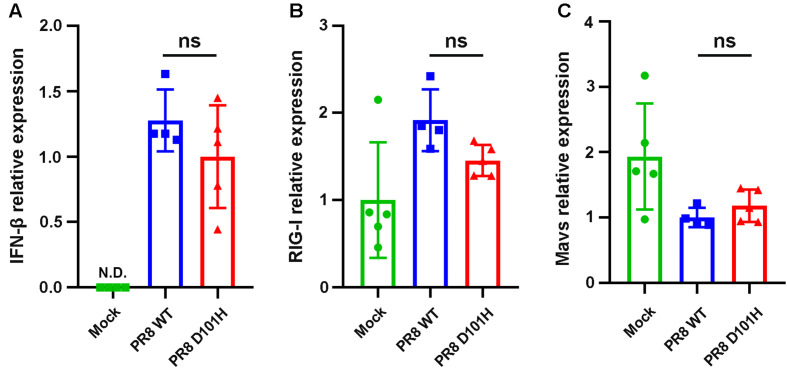
No significant difference in the expression level of innate immune-related genes between the two viruses *in vivo*. Expression of type I IFNs **(A)**, RIG-I **(B)**, and Mavs **(C)** in the lungs of BALB/c mice following influenza infection were determined by qPCR. Lungs were harvested 3 days after infection. Differences between groups were analyzed by unpaired *t*-test (ns, not significant), N.D., not detectable.

We further compared the virus titers of IAV-PR8 and PR8-NS1 (D101H) in the infected lung tissues. No significant differences between the two viruses were observed in the lungs harvested at 3 dpi (Figure 6A). Consistently, immunohistochemical analysis of viral NS1 and M1 proteins revealed no significant differences between the two viruses in the infected lung tissue (Figure 6B). Moreover, RT-qPCR analysis also found similar expression levels of several interferon response pathway genes including type I IFN, RIG-I, and MAVS in the lung tissues after infection with either the wild-type or mutant IAV-PR8 (Figure 7). These results indicate that the single-amino-acid substitution introduced at position 101 of IAV-PR8 NS1 enhanced virus virulence but had no significant effect on virus titers or the expression of key IFN pathway genes.

### NS1 (H101)-Encoding IAV Isolates Are Rare in Nature

**FIGURE 8:**
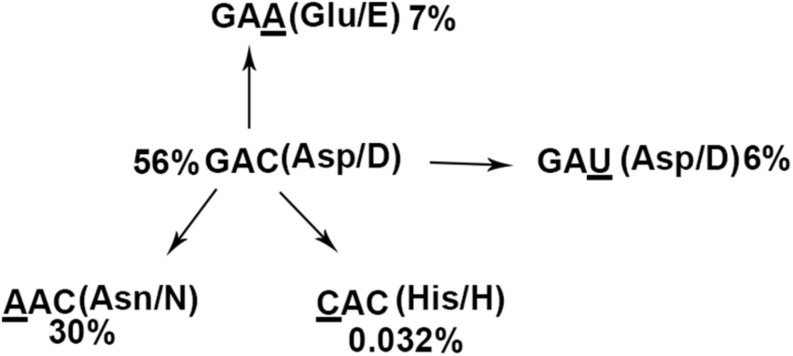
NS1 (H101)-encoding IAV isolates are rare. The pattern of the 101st amino acids and underlying codons of 69,057 influenza NS1 proteins. The dominant codon GAC encoding aspartic acid (56%) is regarded as the ancient codon, whereas GAU (aspartic acid, 6%), AAC (asparagine, 30%), GAA (glutamic acid, 7%), and CAC (histidine, 0.032%) are thought to be derived from GAC with minimal nucleotide mutations.

The NCBI database contained 69,057 entries of NS1 with 100 or more amino acids in length (dated to November 21, 2019) encoded by all circulating subtypes of IAVs isolated worldwide from human and animal hosts (Supplementary Table 1). We found that 43,113 (62%) of these NS1 sequences encode aspartic acid (Asp/D) at position 101 as found in IAV-PR8 by codon GAC (56%) or GAU (6%) (Figure 8). Of all the NS1 entries, 30% and 7% encode asparagine (Asn/N) and glutamic acid (Glu/E) at this position, using codons AAC and GAA, respectively, each of which, similar to GAU, also resulted from a single nucleotide mutation of the dominant codon GAC (Figure 8). Strikingly, only 22 NS1 entries in total, or 0.032% of all NS1 entries, encode histidine (His/H) at position 101 exclusively by codon CAC, as found in IAV-WSN. Thus, codon CAC for H101 of IAV NS1 is significantly underrepresented even though it also differs by a single nucleotide from the dominant codon GAC (Figure 8).

We found that all of the H1N1 viruses isolated in 1933 encode NS1-H101 whereas NS1-H101 is encoded only by one of the 93 H1N1/H7N1/H7N7 viruses identified in the following year. In addition, NS1-H101 was encoded by two of the 7 H1N1 viruses and one of the 3 H1N1 viruses identified in 1935 and 1940, respectively (Supplementary Table 1). Among 23,693 NS1 entries from H3N2 viruses, NS1-H101 is encoded by four isolates collected from California, United States, in 2003 (three isolates) and Hong Kong in 2004 (one isolate). The 16 NS1-H101 protein sequences encoded by H1N1 viruses identified before 1941 share 97–100% identity. Likewise, the 4 NS1 genes of H3N2 viruses isolated after 2002 are identical in both amino acid and nucleotide sequences and encode a histone-like C-terminal tail sequence specific to H3N2 viruses (Supplementary Table 2). However, the NS1-H101 of H3N2 viruses exhibited only 85% amino acid sequence identity with NS1-H101 of IAV-WSN (Supplementary Table 2).

## Discussion

MicroRNAs act as important cytoplasmic regulators of gene expression (Carthew and Sontheimer, 2009; Gebert and MacRae, 2019). However, it is becoming evident that miRNAs also have specific nuclear functions, and some of nucleus-cytoplasm transport mechanisms have also been illustrated (Weinmann et al., 2009; Gagnon et al., 2014; Wei et al., 2014; Kalantari et al., 2016a,b; Liu et al., 2018). In this study, we present the evidence that NS1 of a specific strain of IAV plays a vital role in triggering the translocation of Argonaute-2 and miR-16 from the cytoplasm to the nucleus. Both IAV-WSN and IAV-PR8 NS1 proteins can suppress Dicer processing of viral dsRNA into viral siRNAs in human cells (Li et al., 2017), suggesting that the observed difference may be unrelated to the RNAi suppressor activity of NS1. We propose that the changes induced by IAV-WSN NS1 may disrupt the normal physiological balance of the RISC complex and cause various pathological disorders. Moreover, recent studies implicate miRNAs in the regulation of transcription and splicing in the nucleus (Kalantari et al., 2016a; Liu et al., 2018), suggesting potential modification of the nuclear miRNA function by NS1 of IAV-WSN. In addition, the known cytoplasmic function of AGO2 in antiviral RNAi (Guo et al., 2019) suggests that AGO2 nuclear sequestration by IAV-WSN NS1 may promote virus infection. However, IAV-PR8 and IAV-PR8/NS1 (D101H) replicated to similar levels in lung tissues, indicating that such an effect on antiviral RNAi, if any, is undetectable in the mouse model.

The pathogenicity of influenza viruses is determined by various factors, including specific amino acid mutations of some viral proteins (Fukuyama and Kawaoka, 2011; Tscherne and Garcia-Sastre, 2011; Ayllon and Garcia-Sastre, 2015b). For example, a single amino acid substitution at position 184 of NP protein increases the replication and the pathogenicity of highly pathogenic H5N1 influenza virus in chickens (Wasilenko et al., 2009). A single Glu-to-Lys substitution at position 627 of PB2 protein allows the influenza virus to infect mammalian cells more effectively (Steel et al., 2009; Yamamoto et al., 2013) and Asn at position 701 of PB2 protein is involved in the pathogenicity of the influenza virus in mice (Li et al., 2005; Steel et al., 2009). Moreover, Ser at position 42, glutamic acid at position 92, and C-terminal residues of NS1 protein all could significantly increase the viral virulence *in vivo* (Seo et al., 2002; Lipatov et al., 2005; Jackson et al., 2008; Jiao et al., 2008). In the current work, we show that histidine at position 101 of IAV NS1 plays a unique role to enhance nuclear accumulation of RNAi factors and contributes to viral pathogenesis in mice by a mechanism unlikely to associate with interferon induction or viral replication.

IAVs are evolutionarily dynamic viruses exhibiting high variability in nucleotide and protein sequences. The evolution of IAVs is mainly mediated through antigenic drift and antigenic shift (Wright et al., 2013). Antigenic drift often occurs as a result of point mutations in viruses and refers to minor, gradual, antigenic changes in the HA or NA proteins (Taubenberger and Kash, 2010; Yasuhara et al., 2019). Polymorphism analysis of HA sequence from the past five H7N9 epidemic waves in China showed different serum escape mutations emerged in different waves (Chang et al., 2019). S200P and D144E mutations of HA reported in the 1918 H1N1 viruses can increase the virulence of pHIN1 virus in mice. Only one HA sequence carried both mutations, according to the analysis of H1N1 HA protein from 2009 to 2017 (Al Khatib et al., 2018).

Our bioinformatic analysis combined with a survey of historical records in the NCBI database identifies NS1-H101 as a target for negative selection during the evolution of IAV. Our findings indicate that H1N1 viruses encoding NS1-H101 may be common in 1933 and disappeared completely after 1940. It is well known that viral particle surface proteins are targeted by natural selection to drive the antigenic drift and antigenic shift of seasonal and pandemic strains of IAV (Wright et al., 2013). Our study likely provides evidence for negative selection directed against a viral non-structural protein, which functions to antagonize antiviral responses mediated by both type I interferons and RNAi (García-Sastre et al., 1998; Li et al., 2004, 2017; Kennedy et al., 2015). It is interesting that four of the 23,693 H3N2 viruses isolated after 2002 encode the same NS1 gene with His at position 101. Because of significant sequence variation with NS1-H101 of H1N1 viruses, it is currently unclear if H101 of H3N2 NS1 also plays a role to alter nuclear accumulation of RNAi factors or regulate viral pathogenesis *in vivo*. However, it should be pointed out that only 17 NS1 genes have been sequenced from H1N1 and H7N7 viruses between 1902 and 1931, including a single 1918 isolate (Basler et al., 2001), and none of them encodes the NS1 gene with H101. Thus, it remains to be determined if NS1-H101 is encoded by H1N1 viruses circulating earlier than 1933 such as the highly pathogenic strains of 1918.

## Data Availability Statement

The original contributions presented in the study are included in the article/Supplementary Material, further inquiries can be directed to the corresponding authors.

## Ethics Statement

The animal study was reviewed and approved by all the animal experiments in China were carried out under the guidelines of the Institutional Animal Care and Use Committee, Fudan University of China.

## Author Contributions

HW, ZT, and YL performed all the infection experiments. YX performed the bioinformatics analyses. QW assisted with infection experiments in mice. YL and S-WD conceived the study and designed the experiments. YL, S-WD, and HW wrote the final manuscript. All authors contributed to the article and approved the submitted version.

## Conflict of Interest

The authors declare that the research was conducted in the absence of any commercial or financial relationships that could be construed as a potential conflict of interest.

## Footnotes

1 https://www.ncbi.nlm.nih.gov/genomes/FLU/Database/nph-select.cgi

## Supplementary Material

Supplementary Figure 1 Immunofluorescent analysis of 293T cells ectopically expressing Flag-AGO2 with IAV-WSN infection at 12 h post-incubation. Arrows in the merged image indicate the co-localization of Flag-AGO2 and viral NS1 protein. FLAG-tagged epitope representing AGO2 labeling with AlexaFluor488 (Green). Viral NS1 labeling with AlexaFluor633 (Red). Nuclei counterstaining with DAPI (Blue).

Supplementary Figure 2 Comparing PR8 7 + 1 with IAV-WSN and IAV-PR8. Western blotting detection of RNAi protein components in nuclear and cytoplasmic extracts isolated from 293T cells 12 h after inoculation with buffer (Mock), WSN, PR8, and PR8 7 + 1 viruses. β-actin as loading controls.
