# Supplementary material for: Altering Intracellular Localization of the RNA Interference Factors by Influenza A Virus Non-structural Protein 1
Source: Front Microbiol. 2020 Nov 12;11:590904. doi: 10.3389/fmicb.2020.590904 (PMC7688628; doi:10.3389/fmicb.2020.590904)
Supplement: Supplementary Figure 1 — Immunofluorescent analysis of 293T cells ectopically expressing Flag-AGO2 with IAV-WSN infection at 12 h post-incubation. Arrows in the merged image indicate the co-localization of Flag-AGO2 and viral NS1 protein. FLAG-tagged epitope representing AGO2 labeling with AlexaFluor488 (Green). Viral NS1 labeling with AlexaFluor633 (Red). Nuclei counterstaining with DAPI (Blue). [file Image_1.pdf]

SUPPLEMENTAL MATERIAL

Supplementary Figure S1-2 and Supplemental Tables 1-2

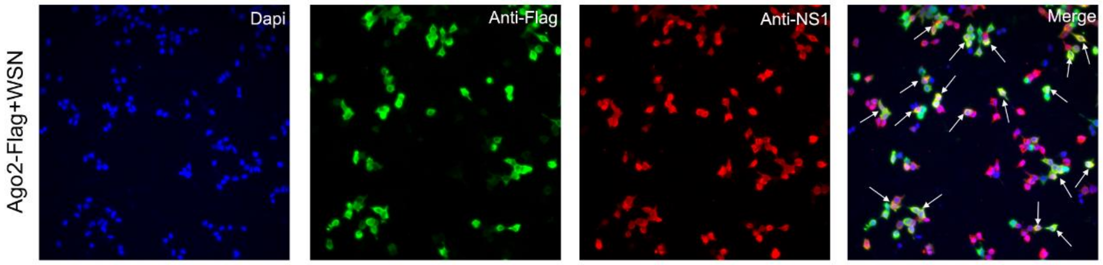

Supplementary Figure S1 Immunofluorescent analysis of 293T cells ectopically expressing Flag-AGO2 with IAV-WSN infection at 12 hours post-incubation. Arrows in the merged image indicate the co-localization of Flag-AGO2 and viral NS1 protein. FLAG-tagged epitope representing AGO2 labeling with AlexaFluor488 (Green). Viral NS1 labeling with AlexaFluor633 (Red). Nuclei counterstaining with DAPI (Blue).

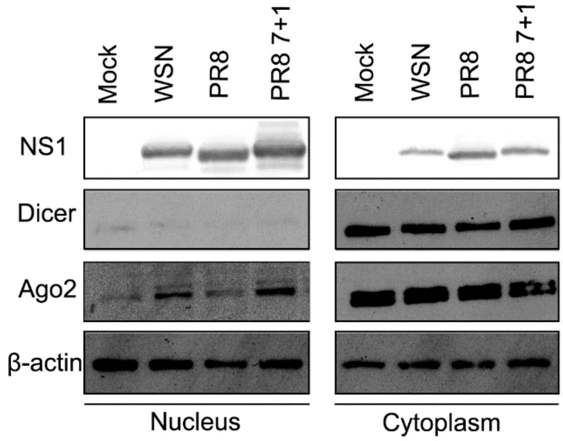

Supplementary Figure S2 Comparing PR8 7+1 with IAV-WSN and IAV-PR8. Western blotting detection of RNAi protein components in nuclear and cytoplasmic extracts isolated from 293T cells 12 hours after inoculation with buffer (Mock), WSN, PR8, and PR8 7+1 viruses. β-actin as loading controls.
