# Supplementary material for: Altering Intracellular Localization of the RNA Interference Factors by Influenza A Virus Non-structural Protein 1
Source: Front Microbiol. 2020 Nov 12;11:590904. doi: 10.3389/fmicb.2020.590904 (PMC7688628; doi:10.3389/fmicb.2020.590904)
Supplement: Supplementary file 3 [file Table_2.DOCX]

Supplementary Table 2: Information of 22 H101-NS1 and their identity to one of the WSN sequences(ACF54603).

| Query | Reference | Identity Score | Serotype | Country | Date | Name |
| --- | --- | --- | --- | --- | --- | --- |
| QDQ43394 | ACF54603 | 100 | H1N1 | Russia | 2016 | A/Moscow/rWSN/2016 |
| QDQ43406 | ACF54603 | 100 | H1N1 | USA | 2000 | A/USA/rWSN/2000 |
| ABF47960 | ACF54603 | 100 | H1N1 | LAB^1^ | 1933 | A/WSN/1933 TS61(H1N1) |
| ACF54603 | ACF54603 | 100 | H1N1 | LAB^1^ | 1933 | A/WSN/1933(H1N1) |
| ABF83571 | ACF54603 | 100 | H1N1 | LAB^1^ | 1933 | A/WSN/1933(H1N1) |
| BBB04706 | ACF54603 | 100 | H1N1 | LAB^1^ | 1933 | A/WSN/1933(H1N1) |
| AAA21580 | ACF54603 | 100 | H1N1 | United Kingdom | 1940 | A/WSN/1940(H1N1) |
| ABD77801 | ACF54603 | 100 | H1N1 | United Kingdom | 1933 | A/Wilson-Smith/1933(H1N1) |
| ABF21218 | ACF54603 | 100 | H1N1 | United Kingdom | 1933 | A/Wilson-Smith/1933(H1N1) |
| AGQ47745 | ACF54603 | 99.565 | H1N1 | NA^2^ | 1935 | A/BH/JY2/1935(H1N1) |
| AEG64767 | ACF54603 | 99.565 | H1N1 | United Kingdom | 1933 | A/United Kingdom/1-MA/1933(H1N1) |
| AAA21582 | ACF54603 | 99.565 | H1N1 | United Kingdom | 1933 | A/WS/1933(H1N1) |
| AEM60009 | ACF54603 | 99.565 | H1N1 | NA^2^ | 1935 | A/bh/1935(H1N1) |
| ACV49539 | ACF54603 | 99.13 | H1N1 | United Kingdom | 1933 | A/United Kingdom/1/1933(H1N1) |
| AAA91330 | ACF54603 | 98.696 | H1N1 | United Kingdom | 1933 | A/NWS/1933(H1N1） |
| AFM68959 | ACF54603 | 98.696 | H1N1 | LAB^1^ | 1934 | A/NWS/1934(H1N1) |
| QDQ43418 | ACF54603 | 98.696 | H1N1 | United Kingdom | 1933 | A/mouse/United Kingdom/WSN/1933 |
| BAQ25795 | ACF54603 | 98.261 | H1N1 | LAB^1^ | 1933 | A/NWS/1933(H1N1) |
| AEG65323 | ACF54603 | 85.652 | H3N2 | USA | 2003 | A/California/NHRC0002/2003(H3N2) |
| AEG65257 | ACF54603 | 85.652 | H3N2 | USA | 2003 | A/California/NHRC0003/2003(H3N2) |
| AEG65312 | ACF54603 | 85.652 | H3N2 | USA | 2003 | A/California/NHRC0004/2003(H3N2) |
| ACO94930 | ACF54603 | 85.652 | H3N2 | Hong Kong | 2004 | A/Hong Kong/HKU9/2004(H3N2) |

1. Sequences of reassortments or lab strains (those flagged as "LAB" in the country field)
2. Not available
